# How to Advertise

**Published:** 1887-03

**Authors:** 


### HOW TO ADVERTISE.

MEDICUS.—Say, Gough! don't you want a little medical item for your daily?

REPORTER.—Hallo, Doc.! To be sure I do; what do you know?

MEDICUS.—Nothing much, except that I have just returned from the Executive mansion, where I spent the whole night.

REPORTER.—What 's up?

MEDICUS.—The Governor's child is very low with diphtheria. I was called in a consultation, and forced to remain with my colleagues.

REPORTER.—Is that so? How many doctors were there?

MEDICUS.—Six. You may state in your journal that they unanimously adopted my plan of treatment. I expect to see the case again this evening. Call again for further details. Have a Key West?

REPORTER.—Thanks. It 's a pity you fellows can 't advertise. But, Doc., whenever you have an item just let me know, and I 'll do the square thing. Bye, bye!

MEDICUS.—More anon. Return this evening.

REPORTER.—I 'll see you later.

